# Glycaemic control and avenues for improvement among people with type 2 diabetes mellitus from rural Sri Lanka – a retrospective cohort study

**DOI:** 10.1016/j.lansea.2023.100169

**Published:** 2023-02-27

**Authors:** Chamila Mettananda, Umesh Chathuranga, Tharanga Rathnayake, Nathasha Luke, Neranjala Meegodavidanage

**Affiliations:** a Department of Pharmacology, Faculty of Medicine, University of Kelaniya - Ragama, Sri Lanka; b South Asian Clinical Research (SACR), T.H. Anuradhapura, Sri Lanka; c Base Hospital, Medawachchiya, Sri Lanka; d Teaching Hospital, Anuradhapura, Sri Lanka

**Keywords:** Diabetes mellitus, Glycaemic-control, Rural, South-Asia, Sri Lanka, Cohort study

## Abstract

**Background:**

The majority of Sri Lankans and South Asians are rural dwellers but follow-up data on glycaemic control and its associations in rural communities are sparse. We followed up a cohort of hospital-based rural Sri Lankans with diabetes from diagnosis up to 24-months.

**Methods:**

We conducted a retrospective cohort study of people with type-2 diabetes (T2DM) diagnosed 24 months before enrolment who were being followed up at Medical/Endocrine clinics of five hospitals selected by stratified random sampling in Anuradhapura, a rural district of Sri Lanka from June 2018 to May 2019 and retrospectively followed them up to the diagnosis of the disease. Prescription practices, cardiovascular risk factor control and their correlates were studied using self-administered and interviewer-administered questionnaires and perusing medical records. Data were analysed using SPSS version-22.

**Findings:**

A total of 421 participants [mean age 58.3 ± 10.4 years, female 340 (80.8%)] were included in the study. Most participants were started on anti-diabetic medications in addition to lifestyle measures. Of them, 270 (64.1%) admitted poor dietary-control, 254 (60.3%) inadequate medication-compliance and 227 (53.9%) physical inactivity. Glycaemic control was assessed mainly on fasting plasma glucose (FPG) and glycated haemoglobin (HbA1c) data were available in only 44 (10.4%). Target achievements in FPG, blood pressure, body mass index and non-smoking at 24-months following initiation of treatment were 231/421 (54.9%), 262/365 (71.7%), 74/421 (17.6%) and 396/421 (94.1%) respectively.

**Interpretation:**

In this cohort of rural Sri Lankans with type-2 diabetes mellitus, all were started on anti-diabetic medications at the diagnosis, but glycaemic target achievement was inadequate at 24 months. We identified the major patient-related reasons for poor blood glucose control were poor compliance with diet/lifestyle and/or medications and misconceptions about antidiabetic medications.

**Funding:**

None.

**Research in context**

**Evidence before this study**

We searched PubMed and Google scholar for articles published between Jan 01, 2001, and Nov 06, 2022 using the terms “glycemic control”, “sugar control”, “type 2 diabetes mellitus”, “Sri Lanka”, and “south Asia” that evaluated blood glucose control in people with type 2 diabetes from Sri Lanka or south Asia. We confined our search to articles published in the English language. The search identified literature exploring the level of blood glucose control only or the effect of specific different interventions like diet, exercise, or knowledge of diabetes self-care on blood glucose control separately in different populations. However, except for one study from Bangladesh, there were no studies looking at glycaemic control and all the reasons for poor glycaemic control in south Asians or Sri Lankan cohorts. Furthermore, there were no descriptive studies on the extent of blood glucose control in rural Sri Lanka.

**Added value of this study**

To the best of our knowledge, this is the first study to examine the reasons behind poor blood glucose control among Sri Lankan people with diabetes mellitus. This study evaluated glycaemic control of 421 individuals diagnosed of diabetes mellitus 2 years prior to recruitment to the study, attending outpatient clinics of all the types of state sector hospitals of a rural district of Sri Lanka. We observed that almost all participants were started on pharmacological management of diabetes mellitus, but only half of the participants had blood glucose controlled to target. The major reasons identified for poor blood glucose control were poor adherence of participants to prescribed treatment options and the suboptimal effort put in by the people with T2DM and physicians to control risk factors to target levels. Poor patient engagement in the management of their illness associated with the south Asian culture was an important factor while lack of up-to-date knowledge of physicians on the latest management options of diabetes and overcrowding of clinics also played important roles.

**Implications of all the available evidence**

Our results indicate that the main effort would be in getting the active involvement of people in the management of their disease. The existing South Asian culture of people, “just consuming whatever is prescribed by the physicians” needs to be changed and the public should be empowered to involve in the management of their health. Encouraging physicians to control diabetes and cardiovascular risk factors to achieve targets according to an individual's total cardiovascular risk also is a priority but will need to address the overcrowding of clinics for this to be practical. While the results are from Sri Lanka, they are likely relevant to other South Asian countries in the region sharing similar cultural backgrounds, overcrowding and per capita incomes, which are a few root courses for poor glycaemic control in the region.

## Introduction

Diabetes mellitus is the most common metabolic disorder in the world^1^ with a global prevalence of 9.3% (as of 2019). The prevalence is higher in urban (10.8%) than rural (7.2%) areas, and in high-income (10.4%) than low-income countries (4.0%).^1^

Diabetes has become an epidemic in low-income and middle-income countries including South Asia due to rapid urbanisation and genetic predisposition.^1^, ^2^, ^3^ Of the world's diabetic population, 60% are Asians and 8.8% among 20-79-year-olds.^2^^,^^4^^,^^5^ South Asians are at increased risk of developing diabetes, developing the disease at a younger age, and developing diabetic complications; especially cardiovascular, renal, and eye complications.^6^^,^^7^ The average age of diabetes onset in South-East Asians is 10 years earlier than for people of European origin.^8^

The prevalence of diabetes is even higher among Sri Lankans (than the average of Asians) with a national prevalence of 10.3%, and 18.6% in the urban parts (Western province).^5^^,^^6^ Diabetes is the second most common non-communicable disease in Sri Lanka, only second to cardiovascular diseases, and is rapidly increasing in prevalence.^7^ In addition, Sri Lanka has a higher diabetes endemicity index (2005/2006–52.8%) compared to other south Asian countries ((rural India 2007–26.9%; urban India 2002/2005–31.3%, and urban Bangladesh −33.1%)).^9^

The majority of South Asians (65.12% in 2020) are from rural settings^7^ and it is the same in Sri Lanka (77% in 2020).^8^ Therefore, addressing diabetes control in rural South Asia is very important. However, most diabetes-related reports from South Asia and Sri Lanka are from urban or semi-urban populations and reports on diabetes management in rural populations are sparse. Furthermore, follow-up data of individual people with T2DM in real life is very low.

Reducing the burden of diabetes in South Asia is a challenge to the world. Studies based on real-life evidence and experience are a practical way of identifying possible areas for improvement. Even though several new therapies for type-2 diabetes have become available leading to a transitional shift in type-2 diabetes management, the recommendations have to be practical, affordable, and specific for South Asians to be cost-effective.^10^

We studied a cohort of people with diabetes attending state hospital clinics in a rural district of Sri Lanka to understand the extent of diabetes control and the main issues related to the management of diabetes to recommend avenues for improvement in diabetes control in rural South Asians.

## Methods

**Fig. 1:**
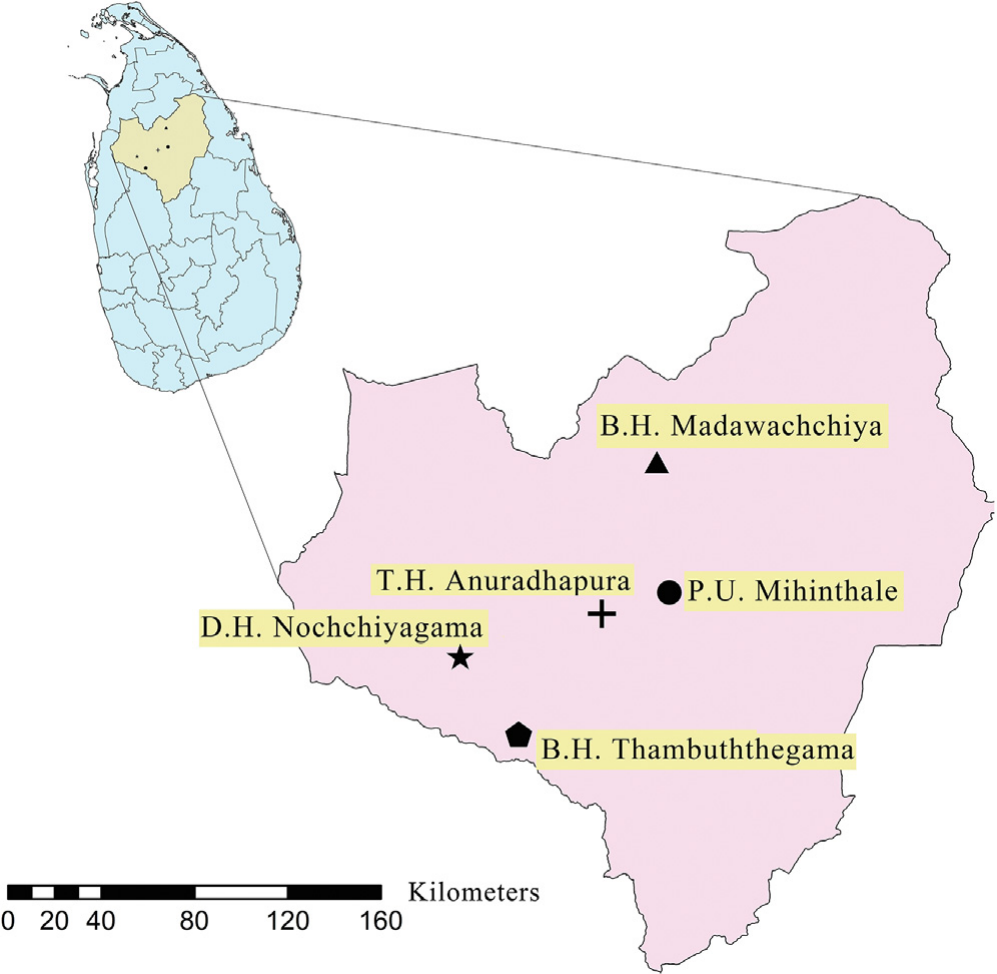
**Study sites.** TH – Teaching Hospital, BH – Base Hospital, DH–District Hospital, PU – Peripheral Unit.

We conducted a retrospective cohort study of people with type 2 diabetes mellitus (T2DM), diagnosed with diabetes 24 months before enrolment, attending medical/endocrine clinics of five randomly selected hospitals (stratified by the hierarchies of hospitals) representing all hospital types (one teaching hospital, two base hospitals, one district hospital and one peripheral unit) in Anuradhapura, a rural district of Sri Lanka from August 2018 to May 2019 (Fig. 1). Data were collected by a post-graduate trainee in Clinical Pharmacology.

All consecutive, consenting, eligible people with T2DM, diagnosed 24 months before index clinic visit attending study clinics were enrolled on the study, following obtaining informed written consent. Participants were enrolled from 5 hospitals, serially, over 10 months. Enrolment of study participants in one hospital was carried out over 2 months. Pregnant mothers, people with Type 1 diabetes mellitus and those with diabetes diagnosed before 24 months or after 24 months were excluded from the study. Data on anthropometry, anti-diabetic medication prescription, glycaemic and other risk factor control over the past 24 months since diagnosis were collected retrospectively using interviewer-administered questionnaires and medical records. This included fasting plasma glucose (FPG) values, blood pressure, lipid measurements and BMI at 0, 3, 6, 9, 12, 15, 18, 21, and 24 months from the diagnosis up to the enrolment in the study. The weight and height of the study participant were measured at enrolment in the study using standard procedures and BMI was calculated. Blood pressure (BP) was measured using an Omron 705CP automated blood pressure monitor in the left arm in the seated position. The mean of two BP measurements, taken 5 min apart was defined as BP. Data on diabetic complications, adverse drug reactions, compliance with diet, exercise and medications were collected using interviewer-administered questionnaires. Participants’ knowledge and awareness of diabetes mellitus were collected using a self-administered questionnaire consisting of five main parts assessing awareness of the disease, disease-related complications, treatment, lifestyle management and risk factors. All current smokers and those who quit smoking less than one year before the assessment were considered smokers.^11^ Overweight was defined as BMI = 23–27.49 kg/m^2^ and obesity was defined as having BMI >27.5 kg/m^2^ according to WHO guidelines.^12^ Target achievement in relation to different cardiovascular risk factor control were defined on local guidelines as, FPG <130 mg/dL, systolic blood pressure <130  mmHg, diastolic blood pressure <85 mmHg, LDL <100 mg/dL and BMI <23 kg/m^2^.^13^

Data were analysed using SPSS version 22. The significance level was set at p < 0.05. Shapiro–Wilk test was used to check normality distribution. Descriptive statistics were used to compare variables at the two-time points; i.e: at the diagnosis and 24 months. Means of continuous variables were compared using paired t-tests. Mean FPG levels achieved at 0, 3, 6, 12 and 24 months since diagnosis of diabetes mellitus were compared between the anti-diabetic medication prescription regimen at the initiation of treatment (ie: mono-vs multi-drug therapy) using linear regression. Categorical variables were compared using the χ^2^ test. Associations to the prescription practices; mono-vs multi-drug therapy was studied using logistic regression. Association to age, BMI, sex, smoking status, presence of hypertension and hyperlipidaemia and being treated in a consultant lead clinic, presence of diabetic complications on diagnosis and mean FPG at baseline were studied individually.

### Role of the funding source

Not applicable.

## Results

A total of 421 participants [mean age 58.3 ± 10.4 years, female 340 (80.8%)] from five sites were recruited for the study. The demographics of the study population are shown in Table 1. The majority of participants were Sinhalese [415 (98.6%)] females (340 (80.8%)). Family history of diabetes in first-degree relatives was present in 183 (43.5%) participants.

**Table 1:** Demographics of the study participants.

Variable	N = 421 (%)
**No of participants per site**	
Teaching Hospital Anuradhapura	226 (53.7%)
Base Hospital Madawachchiya	153 (36.3%)
Base Hospital Thambuththegama	21 (5.0%)
District Hospital Nochchiyagama	10 (2.4%)
Peripheral Hospital Mihinthale	11 (2.6%)
Mean age (SD) years	58.3 ± 10.4
Female sex n (%)	340 (80.8%)
**Ethnicity**	
Sinhalese	415 (98.6%)
Other	6 (1.4%)
**Educational level**	
Not schooled	147 (34.9%)
Primary	233 (55.3%)
Secondary	37 (8.8%)
Tertiary	4 (1.0%)
**Occupation**	
Professional	183 (43.5%)
Semi-professional	12 (2.9%)
Clerical work	33 (7.8%)
Skilled worker	21 (5.0%)
Non-skilled worker	173 (41.1%)
Unemployed/housewife/retired	182 (43.2%)
Family history of T2DM in first-degree relatives	183 (43.5%)

T2DM - type 2 diabetes mellitus.

### Prevalence of metabolic and cardiovascular risk factors

Prevalence of metabolic and cardiovascular risk factors at the diagnosis of diabetes and 24 months after the diagnosis were compared using the χ^2^ test and is shown in Table 2. A statistically significant reduction in the prevalence of obesity (p < 0.001) but an increasing prevalence of overweight (p < 0.001) was observed. There was no change in the prevalence of smoking or hypertension observed. Mean FPG (p < 0.001), systolic blood pressure (p = 0.010), and BMI (p < 0.001) showed significant positive improvements.

**Table 2:** Comparison of baseline cardiovascular and metabolic risk factors at diagnosis and 2 years after diagnosis of diabetes mellitus.

Variable	At diagnosis	24 months following diagnosis	p
	N = 421 (%)		
**Cardiovascular risk factors**			
Smoking	20 (4.7%)	25 (5.9%)	0.06
Hypertension	207 (49.1%)	208 (49.4%)	1.00
Dyslipidaemia	260 (61.7%)	262 (62.2%)	0.50
Overweight	131 (31.1%)	203 (48.2%)	<0.001
Obese	194 (46.1%)	144 (34.2%)	<0.001
Physical activity <150 min/week	…	186 (44.1)	…
**Measured risk factors**			
Systolic BP (mean ± SD) mmHg	159.7 ± 21.0	147.6 ± 13.4	0.01
Diastolic BP (mean ± SD) mmHg	93.0 ± 13.4	88.3 ± 7.3	0.05
FPG (mean ± SD) mg/dL	224.5 ± 87.1	138.6 ± 54.38	<0.001
BMI (mean ± SD) kg/m^2^	27.4 ± 5.9	26.5 ± 4.5	<0.001

FPG - Fasting plasma glucose, BP - blood pressure.

### Anti-diabetic medication prescription practices

**Fig. 2:**
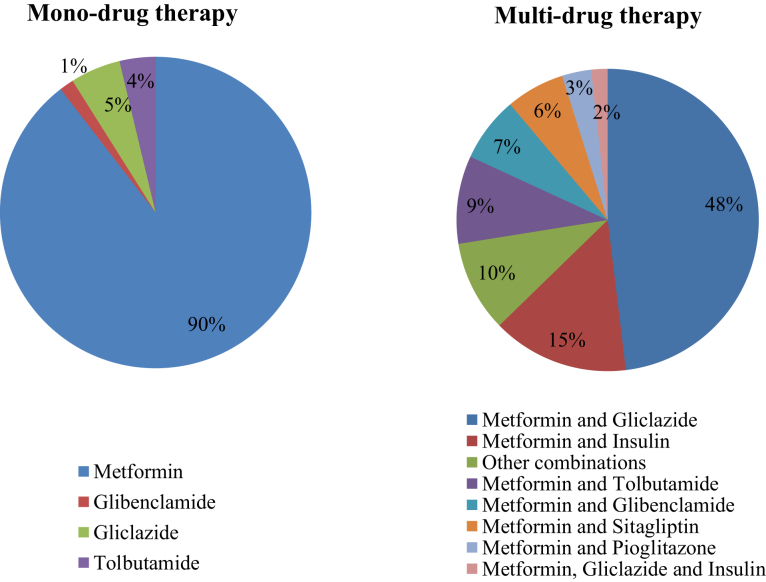
**Anti-diabetic medicationprescription at the initiation of treatment for type 2 diabetes mellitus; mono-therapy vs multi-drug therapy regimen.**

All people withT2DM were prescribed anti-diabetic medications in addition to dietary management, and none was put solely on diet control. The groups of antidiabetic medications initiated at the diagnosis of T2DM are shown in Fig. 2. The majority 287 (68.2%) were started on multi-drug therapy.

Metformin was the most prescribed drug, in both mono-drug therapies (89.6%) and multi-drug therapy (98.5%) prescriptions. The most prescribed multi-drug combination was metformin and gliclazide in 138 (48%). Insulin was started along with oral medications in 23 (5.4%) participants at the initiation of medications which rose to 42 (17%) at the end of two years. None of this rural population was started on sodium-glucose transport protein 2 (SGLT2) inhibitors or Glucagon-like peptide 1 (GLP1) agonists. Over the 24 months, 32 (23.8%) participants on mono-drug therapy were escalated to multi-drug therapy.

The factors associated with the decision on starting multi-drug therapy compared to mono-drug therapy at the initiation of medications for diabetes mellitus were, having high baseline FPG (p < 0.001), diabetic complications at the diagnosis of diabetes (p = 0.004) and being treated in a consultant/specialist-led clinic (p < 0.002) (Table 3).

**Table 3:** Factors associated with the initiation of multi-drug therapy over mono-drug therapy at the initiation of treatment for type 2 diabetes mellitus.

Factors	Monotherapy	Multi-therapy	P^a^
	n = 134	n = 287	
	31.82%	68.17%	
Mean age (SD) years	54.8 ± 11.5	55.11 ± 9.7	0.96
Mean BMI (SD) kg/m^2^	26.0 ± 4.7	26.7 ± 4.5	0.17
Male sex	22 (16.7)	57 (19.9)	0.50
Current Smoking	7 (5.3)	13 (4.5)	0.81
Coexistent hypertension	63 (47.7)	144 (50.3)	0.67
Coexistent hyperlipidaemia	88 (66.7)	172 (60.1)	0.23
Treated by a Consultant lead clinic	73 (55.3)	108 (37.8)	0.002
Presence of diabetic complications at the diagnosis of diabetes	18 (13.3)	76 (68.4)	0.004
Mean FPG at baseline (mg/dL)	193.6 ± 72.5	238.5 ± 89.7	<0.001

FPG - Fasting plasma glucose.

a Un-adjusted p values in univariate logistic regression.

### Glycaemic control

FPG was the main parameter used to assess glycaemic control in this population and almost all 415 (98.6%) have had FPG done at least once every 2 months. However, FPG checking facilities were not available in peripheral units and the participants had to get those done from the private sector. HbA1c testing facilities were available only in the Teaching hospital, Anuradhapura. However, only 44 (19.5%) out of 226 participants enrolled on the study from the said hospital have had at least one HbA1c test done over the 24-month follow-up period.

**Fig. 3:**
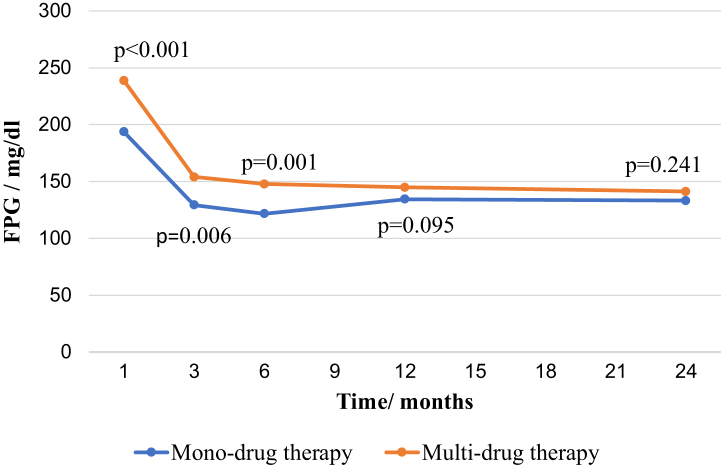
**Time trends in the mean fasting plasma glucose over 24 months with mono- or multi-drug therapy.** FPG - Fasting plasma glucose. P – p values for the differences between mean FPG of participants on mono-drug therapy and multi-drug therapy after adjusting for “treated by consultant lead clinics” and “FPG at baseline”.

The time trends in the mean fasting plasma glucose values of the population who were started on mono and multi-drug therapy over the 24 months are shown in Fig. 3. We randomly selected hospitals of each stratum to get a fair representation of all types of hospitals, but we did not stratify by 'hospital type' in the analysis. However, we adjusted the data for being treated at a consultant-led clinic or non-consultant-led clinic in the multivariate model used in Fig. 3. Of the four types of hospitals included in the study, the teaching hospital and the two base hospitals were consultant-led, and the district hospital and the peripheral unit were non-consultant-led.

Mean FPG values adjusted for being treated at consultant lead clinics and baseline FPG at 3-months or 6-months following initiation of treatment were significantly different between monotherapy and multi-drug therapy groups (p = 0.006, p = 0.001 respectively), but this difference gradually disappeared by the end of 12 months after initiation of medications and remained statistically not significant thereafter (p = 0.095, p = 0.241 respectively).

However, 190 (45.1%) of the total population had uncontrolled FPG levels at the end of 24 months of treatment.

### Target achievement in other cardiovascular and metabolic risk factors

Cardiovascular risk factors control according to local guidelines showed the following. BP < 130/85 mmHg in 262/365 (71.7%), BMI <23 kg/m^2^ in 74/421 (17.6) and non-smoking state in 396/421 (94.1%). Of the people aged 40–70 years, 250/377 (66.3%) were prescribed statins. However, most participants had not had lipid profiles checked despite facilities for checking lipid profiles being available in all the hospitals except peripheral units. Of the 97 who had data on LDL cholesterol levels, only 29.9% had LDL <100 mg/dL. Of all, 46 (10.9%) were on aspirin at the 24-month follow-up.

### Adverse drug reactions

Gastrointestinal disturbances (nausea, vomiting, diarrhoea and loss of appetite) were the most common side effects experienced by participants with a prevalence of 22.3%. Hypoglycaemia was reported by 105 (24.9%) participants; in 77/379 (18.3%) on oral hypoglycaemic drugs only and 28/42 (6.6%) on insulin-based regimes. Lipo-hypertrophy at insulin injection sites was reported by 30/42 (71.4%) participants on insulin. The insulin injection technique was not perfect in almost all 41 (97.6%) participants, and they had been injecting a part of the insulin intramuscularly instead of subcutaneous route. Three participants, who were prescribed insulin, did not have insulin storage facilities in their homes and had to walk to the nearest hospital daily for their injections.

Most participants, 414 (98.3%) were aware of the importance of lifestyle and dietary modifications, but adherence to a therapeutic lifestyle was not adequate as per the target level. Of all, 270 (64.1%) admitted poor dietary control with consumption of refined sugar or sweets on daily basis. Inadequate physical activity defined as less than 150 min of aerobic exercise per week was seen in 227 (53.9%) participants.

Poor drug compliance (self-reported) was seen among 254 (60.3%) of the participants. The major reasons indicated by participants for poor medication compliance were forgetting to take medications [171 (40.6%)], alleviation of previously troublesome symptoms and not feeling like continuing treatment [93 (22.1%)], undue fear of developing drug-related side effects like renal failure [90 (21.3%)] and too complex medication regimens [63 (14.9%)].

### New-onset diabetes related complications at 24-month follow-up

The prevalence of diabetic complications at the diagnosis and the end of 24 months is shown in Table 4. Diabetes-related complications (self-reported and/or confirmed with medical records) were present in 90 (21.3%) participants at the diagnosis of diabetes mellitus. Numbness of the hand or feet was the most common complication disclosed by 61 (14.5%) of participants at the diagnosis and five participants (1.2%) had presented with diabetic ketoacidosis. Half of the male participants complained of impotence at 24 months after diagnosis of diabetes mellitus. Even though all the hospitals except peripheral units have facilities for checking serum creatinine, only 276 (65.6%) have had it checked.

**Table 4:** Prevalence of diabetes-related complications at diagnosis and 24 months following diagnosis.

	At diagnosis N = 421	At 24 months following diagnosis
	N = 421	
***Microvascular complications*** n (%)		
Blurred vision	…	81 (19.2)
Numbness of hands/feet	61 (14.5)	103 (24.5)
Serum creatinine more than the upper limit of normal^a^^,^^c^	…	34 (8.0)
***Macrovascular complications*** n (%)		
Ischaemic heart disease	7 (1.7)	57 (13.5)
Intermittent claudication	0	3 (0.7)
Stroke	4 (0.9)	15 (3.5)
***Metabolic complications*** n (%)		
Hypoglycaemia	0	120 (28.5)
Diabetic ketoacidosis^a^	5 (1.2)	8 (1.9)
Hyperosmolar hyperosmotic state^a^	0	2 (0.4)
***Autonomic complications*** n (%)		
Erectile dysfunction	0	42 (51.8)^b^
***Other complications***		
Recurrent infections (Skin/Lung/Urinary)	0	30 (10.9)

a Data confirmed from medical records.

b Percentage is from the total male population.

c 145 - missing values.

## Discussion

This is a retrospective follow-up of individual people with diabetes mellitus from diagnosis up to 2 years post-diagnosis of a rural population with diabetes mellitus from South Asia and is the first comprehensive description of such from Sri Lanka. Our study describes individual-level data on all the aspects related to diabetes management in a representative sample of rural people with diabetes seeking medical attention from state sector hospitals of Sri Lanka giving a snapshot of real-life experience and the situation. We observed that all people with diabetes who attended medical clinics were started on pharmacological management in addition to diet control. However, only half of them achieved glycaemic targets at the end of two years. The major patient-related reasons identified for poor blood glucose control were poor compliance with diet/lifestyle and/or medications and misconceptions about antidiabetic medications. In addition, factors related to treating physicians and healthcare system-related factors could have contributed to poor glycaemic control in this population. Non-adherence of physicians to treatment guidelines, very short consultation times allocated for one patient at busy outpatient clinics, and non-availability of some newer and efficacious medicines and investigations in the rural hospitals were a few of them.

Poor compliance with diet/lifestyle and medications were among the main reasons identified for poor glycaemic control among most south Asian,^14^, ^15^, ^16^ and the causes were not different in this rural population. This is especially a problem for South Asians due to the cultural and religious differences compared to Western populations. Rural Asians depend mainly on a rice-based staple diet. They spend less time on their well-being and continue to work for children. Rural South Asian females compared to males were less eager to exercise as it is not part of their culture and with the lack of gender-specific exercise facilities. This is reported in previous observed studies.^14^ More than 80% of the participants were overweight or obese which is shocking for South Asians and the majority were using refined sugars and excess starchy foods despite that. Even though traditional rural economies were based on agriculture, it has now changed with time and more people sought to sedentary lifestyles. This paves way for the increase in cardiovascular and metabolic risk factors like physical inactivity, obesity and diabetes leading to a vicious cycle. The situation is the same with other South Asian communities.^2^^,^^17^^,^^18^

Even though poor drug compliance had been identified as a course for poor glycaemic control, the problem of misconceptions about antidiabetic medications was not highlighted as a serious concern before. It is reported that medication non-adherence in South Asians was affected by the participants’ education and occupation status. Dietary noncompliance was influenced by the level of education and place of treatment.^19^ With poor literacy, not knowing the consequences of missing doses, resorting to traditional medicines, and putting less importance on the health with the fight to live is evident by finding that more self-employed people miss more medicine doses than job holders.^19^^,^^20^ Poor awareness of the disease management and the treatment goals in diabetes control could also have contributed to poor glycaemic control as shown by other studies.^21^^,^^22^ The low socio-economic status and non-empowerment and less readiness to engage in diabetes self-care are other possible reasons for poor glycaemic control in this population as evidenced by other studies.^15^

Another important yet worrisome finding was the higher percentage of participants who were using poor technique for administering insulin. This could be easily overcome by proper education of the people with T2DM, but the bottleneck is the overcrowding in clinics.

All people with diabetes were started on antidiabetic medications according to current diabetes management guidelines and this trend is commendable compared to other South Asian countries.^6^ However, despite all participants being started on pharmacological treatment, only a little more than half achieved therapeutic targets. A cross-sectional study among 3,000 urban dwellers with T2DM conducted in National Hospital Sri Lanka also observed the same despite having the best possible care in state sector hospitals of the country.^23^ Sri Lanka has a very good free health care system, with good life expectancy at birth^24^ and up-to-date guidelines in the management of main non-communicable diseases.^25^ The public literacy rate is also high at 92.3%.^26^ Therefore, inadequate glycaemic control among Asians must be contributed to by several other reasons in addition to poor knowledge among the patient population. Physician-related factors are also likely to contribute to this. Minimal use of newer effective anti-diabetic medications and injectables and poor utilization of HbA1c in monitoring glycaemic control may be associated with inadequate up-to-date knowledge of treating physicians in addition to poor availability of those facilities. Fear and unwillingness of people with T2DM to be started on insulin and newer medications are also significant contributors, especially in Asian culture and in rural communities. The unavailability of a regular supply of commonly used medicines at times and some newer medications are some other possible causes of poor glycaemic control. More than all these, the extremely short time spent between medical officers and the people with T2DM due to overcrowding of diabetic/medical clinics is a reason. There is not enough time to titrate medications adequately to achieve the glycaemic targets of individuals. This problem is seen in both urban and rural communities of South Asia. Some of these reasons were discussed in a systematic review on gaps and barriers to achieving glycaemic control In South Asia.^27^

Metformin was the most widely used medication and metformin, gliclazide was the most used dual antidiabetic combination in our study sample. None of the participants was on SGLT2 inhibitors or GLP1 agonists. The possibilities for this finding are the unavailability of these newer expensive medicines in the government sector, the high cost and addressing poor awareness of the physicians about newer hypoglycaemic medications. The prescription practices of oral hypoglycaemic drugs in this rural cohort were not different to the practice at the National Hospital of Sri Lanka which caters for an urban population.^14^ However, it was observed that a fair number of participants were started on sitagliptin as the second-line medication which is not freely available in state hospitals and is expensive to spend on out of pocket. This suggests that the prescription practices are not simply determined by the availability and the cost indicating inadequate awareness of the physicians also plays a part. This had been identified as a barrier to good glycaemic control in South Asia in previous studies^8^ and is an area needing improvement to achieve diabetes treatment targets.

Even though both international and national guidelines recommend checking HbA1C to assess glycaemic control,^27^^,^^28^ only 10.4% of our study population have had HbA1c checked. However, it was performed in only 20% of the people with T2DM attending Anuradhapura teaching hospital where it is freely available. A study of urban people with diabetes from Sri Lanka also reported HBA1c test was done in 59%^23^ where maximum facilities to do the test were available. Only 23.0% of our rural population have had lipid profiles done despite it being available at most hospitals except peripheral units studied. However, it is only 43.2% of the participants in the said urban population have had lipid profiles done indicating the under-utility of available tests. The situation was the same with serum creatinine measurements. All these suggest that the limited availability of facilities is not the sole reason for not practising treatment guidelines. The possibility for under-utilisation of available tests could be due to overcrowding of clinics, but poor awareness or inertia of the physicians may also be playing a role.

However, there is a proportion of people with poor glycaemic control despite both participants and physicians having satisfactory knowledge of diabetes. This is likely to be due to the inertia of both people with T2DM and treating physicians as also observed by the previous researchers.^29^ South Asians, compared to western populations do not partake in self-management of diabetes and depend on what is told by the physician, which is also one important aspect needing improvement.^28^

Almost half of our study participants had a family history of diabetes mellitus. This is in agreement with previous studies reporting Asians having a higher genetic predisposition to T2DM than Europeans.^2^ This also highlights the importance of targeting the public and not only the people with diabetes to reduce the burden of diabetes in Asians. Increasing awareness of primary prevention of diabetes mellitus through conducting awareness programmes targeting the general public and especially the people with family histories of diabetes is a high priority in South Asia.

Finally, good diabetic control does not mean tight glycaemic control only. Managing all cardiovascular risk factors in the total risk approach compared to individual risk factor control is recommended as superior and cost-effective, especially in South Asia.^29^ However, this rural South Asian population of people with diabetes had suboptimal control of FPG, BP, and BMI at 24-months following the diagnosis. These findings were not different to that of an urban population with diabetes in Sri Lanka.^23^

Our study has several strengths. This study gives individual patient data over 24 months and is a follow-up study that allows understanding of the different aspects of the management and outcomes of individual people with diabetes from diagnosis to up to 2 years. This is the very first surveillance data of people with diabetes from rural Sri Lanka. The participants were selected from a stratified sampling of the hospitals in the relevant rural district allowing us to generalize findings to Sri Lankan rural people with diabetes seeking medical care from state hospitals.

However, there are a few limitations to acknowledge in the current study. Of our participants, 81% were females, which could be due to two reasons. First, fewer males attend medical clinics as they were busy being the sole breadwinners of Sri Lankan families, especially in rural communities. Second, more Sri Lankan females are overweight/obese compared to men,^10^ which leads to a higher prevalence of diabetes in females and more females seeking treatment for diabetes. We did not calculate a required sample size before the study, but we studied all the eligible and consenting patients who came to the clinic during the study period. Also, as the data were collected retrospectively, there is a possibility for recall bias. However, we have tried to minimise recall bias by cross-checking all possible data with medical records. Furthermore, prevalence of diabetic complications was based mainly on clinical history as objective measurements were not available in these resource-poor settings. We studied only the people with T2DM attending state hospitals, but a fair number of Sri Lankans are now solely managed at non-state sector clinics and this population is not represented in this study. However, most rural communities are managed by the state sector hospitals and therefore our conclusions are largely generalisable to both hospital-based and community-based rural people with diabetes of Sri Lanka and largely to rural South Asia.

There are several less expensive and low-tech steps that Sri Lanka and other developing countries could adopt to reduce the burden of poorly controlled diabetes. Improving awareness of the people with diabetes about the importance of lifestyle and medication compliance is important while public education regarding how to prevent developing diabetes mellitus is also paramount. Empowering and encouraging people with T2DM to engage in the self-management of diabetes is important. Educating people with T2DM against misconceptions about antidiabetic medications also takes priority. On the other hand, strengthening treating physicians on up-to-date management of diabetes including special training on starting injectables and newer anti-diabetic is also important. Furthermore, reducing the overcrowding of clinics allowing doctors to spend adequate time with one patient and recruiting clinical pharmacists allowing people with diabetes to discuss and resolve their drug-related problems would enhance drug compliance as well as goal achievement. Therefore, in a nutshell, all sectors involved in diabetes management including people with T2DM, treating physicians and support staff, medical administrators, the public, and the Government need to pay collective attention to reduce the burden of the disease on society and individuals.

A community-based study in future would help to identify the problem furthermore. In addition, conducting audits following education programs targeted for both people with T2DM and the public as well as following arranging periodic continuous medical education programs for treating physicians would help to verify the results of the less expensive low-tech recommendations derived of this study.

## Contributors

CM and UC were involved in conceptualization, data curation, formal analysis, investigation, methodology, project administration, resources, validation, visualization, writing – original draft, writing – review & editing. In addition, CM was involved in software and supervision. TR and NM were involved in project administration, resources, visualization, writing – original draft, writing – review & editing. NM was also involved in supervision. NL was involved in validation, visualization, writing – original draft, writing – review & editing. CM and UC have accessed and verified the data and were responsible for the decision to submit the manuscript.

## Data sharing statement

De-identified individual participant data and a data dictionary defining each field in the set can be made available to others on approval of a written request to the corresponding author. The request will be evaluated by a committee formed by a subset of co-authors to determine the research value. A data-sharing agreement will be needed.

## Editor note

The Lancet Group takes a neutral position with respect to territorial claims in published maps and institutional affiliations.

## Declaration of interests

None.
