# Supplementary material for: Glycaemic control and avenues for improvement among people with type 2 diabetes mellitus from rural Sri Lanka – a retrospective cohort study
Source: Lancet Reg Health Southeast Asia. 2023 Feb 27;12:100169. doi: 10.1016/j.lansea.2023.100169 (PMC10305889; doi:10.1016/j.lansea.2023.100169)
Supplement: Supplementary material [file mmc1.docx]

## Title:

Glycaemic control, complications, drug-related adverse effects and compliance with medications among a group of type 2 diabetes patients attending Endocrine clinic and medical clinics Teaching Hospital Anuradhapura**, D.H. Nochchiyagama, D.H. Medawachchiya, P.H. Mihinthale, B.H. Thambuththegama**

Name P.A.D.U. Chathuranga

**ERC No: ERC/2018/22**

PGIM Supervisor : Dr. Chamila Mettananda

Research Supervisor : Dr. Niranjala Meegodawidanage

**Introduction**

Background information:

Diabetes mellitus (DM) is a metabolic disease due to insulin deficiency, resistance to insulin or both(1). According to the WHO estimates the global prevalence of DM in 2014 was 422 million. (2)This is expected to be doubled in 2030 with a great impact on Asia, Africa and South America. (3)Due to the rapid economic development and urbanization people's lifestyle have shifted to a more sedentary pattern. Together with this change, the increase in consumption of unhealthy diets mainly fast foods has led to the development of central obesity leading to DM.(3)

Type 2 diabetes is preceded by an asymptomatic subclinical phase of 4-7 years between actual onset and clinical diagnosis. This is referred to as the pre-diabetes stage. Uncontrolled diabetes mellitus leads to the development of serious complications which are divided into two broad categories macrovascular and microvascular. Macrovascular complications are mainly due to enhanced atherogenesis which includes cerebrovascular disease, cardiovascular disease and peripheral vascular disease which carry significant morbidity and mortality. Microvascular complications of diabetes are mainly due to persistent hyperglycaemia causing retinopathy, nephropathy and neuropathy (1).

The main management goal of diabetes is to prevent getting complications by controlling glycemic levels to near normal values. According to the American Association of Clinical Endocrinologists, fasting blood glucose level should be less than 110mg/dL and two-hour postprandial blood glucose level should be less than 140mg/dl with HbA1c of less than 6.5%. However less stringent criteria apply for those with other co-morbidities and reduced life expectancy. (4)

The first line oral hypoglycemic agent for type 2 diabetes is Metformin unless contraindicated. Metformin has proved to have long term safety, high efficacy with comparatively low cost and cardiovascular risk protection as the pharmacological therapy for diabetes. (5) In addition to that it is recommended for obese people to prevent developing diabetes. (5)However it causes some gastrointestinal disturbances including loss of appetite, nausea and vomiting which some patients find difficult to tolerate

The second line of oral hypoglycaemic agents include sulfonylureas, α glucosidase inhibitors, DPP-4 inhibitors, GLP1 receptor agonists, thiazolidinedione and SGLT-2 inhibitors. Out of these drugs, sulfonylureas have the potential to cause hypoglycaemia which can be serious. Other categories of drugs also have various side effect profiles. Some recently discovered drugs have the additional disadvantage of being very costly. (4)

The last step in managing type 2 diabetes, failing the oral hypoglycaemics is the addition of insulin. Patients need to be well educated and trained in insulin injection techniques to minimize serious complications like hypoglycaemia. (4)

Management of diabetes mellitus always requires a multidisciplinary approach with pharmacological, dietary habits and physical activities playing key roles. One of the key factors for preventing serious undesirable complications is patients’ adherence to their medication and lifestyle changes. This will be important to achieve both good glycemic control of the patient as well as to reduce wastage of healthcare resources. (4) Poor adherence to diabetic treatment is influenced by several factors. These include patient-centred factors such as influence from comorbidities and physical or mental disabilities, therapy-related factors such as multidrug therapy and complicated regimen with drug-related side effects. Other than these things health care system factors such as negative relationships with healthcare providers, disease-related factors such as symptomless disease conditions and socio-economic factors such as lack of social stability are also responsible for negligence and poor adherence to treatment (6)(7). It is well known that the duration of DM, poor control of the disease, hypercholesterolemia and hypertension lead to the development of macro and microvascular complications. (8)

The situation in Sri Lanka

In Sri Lanka, DM is a serious health problem among non-communicable diseases and the national prevalence is estimated to be around 10.3%. (9)Another study has been conducted in all nine provinces of Sri Lanka which has found province and ethnic-specific prevalence. According to that study, the western province has the highest prevalence (18.6%) while Uva province showed the lowest prevalence (6.8%) Interestingly that study has also found that Sri Lankan Tamil ethnic group had the highest prevalence of type 2 diabetes with 22.1%. (10)

The relatively high prevalence of diabetes in Sri Lanka requires urgent intervention to prevent or delay the onset of complications. (11)Moreover several studies have stressed the importance of strict dietary control, involvement in physical exercise and adherence to the medication in the management of diabetes. (12)

Another important risk factor for getting ischemic heart disease is metabolic syndrome. Among type 2 DM patients in Sri Lanka, the prevalence of the metabolic syndrome is estimated to be around 63.7%. (13)Patients who had abnormal waist circumference was73.9% which was the commonest abnormality among metabolic syndrome patients. (13). Apart from that, some complications such as retinopathy (15%), neuropathy (25.1%) and nephropathy (29%)have been present at the time of diagnosis among a group of type 2 diabetes patients in the western province, of Sri Lanka. (14) Complications of DM occur with the time of disease. But exposure to a long term asymptomatic status and poor access to get health care facilities due to low socioeconomic status may be contributory factors to the development of complications at the time of diagnosis of DM.(14)

The College of Endocrinologists of Sri Lanka has issued national guidelines for the management of diabetes. According to that monotherapy with metformin and lifestyle modification including dietary control, increased physical activities and reduction of weight is the first choice for the patients who cannot control glycemic levels only with lifestyle modifications. If the patient presents with FBS > 200 mg/dL at the initial assessment, it is recommended to start with dual therapy. Also if the target is not achieved within approximately after 3 months of initiation with monotherapy, it is recommended to consider a combination of metformin with a second-line drug or with basal insulin. If the glycemic target is not achieved in three months or If a new patient presented with very high FBS>300 mg/dl, the patient should be treated with dual or triple therapy with insulin. It is recommended in the guidelines that early multidrug therapy is preferred over prolonged monotherapy in achieving glycemic targets. (15)

Justification:

In Sri Lanka, very few national studies have been conducted to assess glycemic control, compliance with medications and drug-related adverse effects among DM. (13)(14) Most of the studies have been focused on the western province of Sri Lanka. This study will be conducted in the Anuradhapura district which is situated in the north-central province. The majority of the community in this district is engaged in agriculture-based activities which require significant physical exertion. Also, their monthly income is relatively low and they do not have adequate health education levels compared to the urban population. They have their own beliefs and myths related to disease conditions and treatments and some people give priority to native and homemade remedies for their diseases. Only one study could be found in the literature survey which was conducted island-wide including the Anuradhapura district. According to that study, the prevalence of type 2 diabetes in the north-central province was 9.6%. (10) However this study has mainly focused on the province-specific prevalence of type 2 diabetes and data on glycaemic control and complications are lacking. Therefore this study was planned to assess the glycemic control, compliance with medications, drug-related adverse effects and diabetes-related complications of the patients residing in this area.

**Objectives:**

General objective;

To assess glycemic control of adult diabetes mellitus (DM) patients who are in different levels of hyperglycemia at the time of diagnosis.

Specific objectives;

1. To describe glycemic control of monotherapy vs early multidrug therapy to achieve the glycemic target in adult Type-2 DM patients.
2. To describe the frequency of complications at presentation (macrovascular/ microvascular/ short term)
3. To describe oral hypoglycemic medication-related adverse drug reactions and tolerability.
4. To identify the degree of drug compliance and reasons for non-compliance with medications.

**Method**

**Study design and setting**

This will be a cross-sectional study conducted in outpatient diabetes and endocrinology clinic, other medical clinics, Teaching Hospital, Anuradhapura, D.H. Nochchiyagama, D.H. Medawachchiya, P.H. Mihinthale, B.H. Thambuththegama, C.D. Secret City, P.H. Thalawa and P.H. Parasangaswewa. All the consenting type 2 DM patients diagnosed (criteria for diagnosis of type 2 DM, 10 hours fasting blood glucose (FBS) ≥126mg/dL or ≥7.0mmol/L and HbA1c is >6.5%) and under anti-diabetes therapies for at least 24 months at the time of enrollment will be included in this study. Patient details will be collected retrospectively using medical records and interviewing patients concerning 4 defined periods at the time of diagnosis, 3 months, 6 months, 1 year and 2 years after diagnosis.

Data collection will be carried out within 6 months period from June to November 2018 (Annexure I). Pregnant mothers, age <18 years and Type 1 diabetes patients will be excluded. This study will be conducted after obtaining both ethical clearance, from the ethical review committee of the Rajarata University of Sri Lanka and the approval from the Director, Teaching hospital, Anuradhapura. Verbal and informed written consent will be obtained from the study patients before enrolment. The consent form and information sheet are formulated in all three languages including Sinhala, Tamil and English (Annexures I, II, IV). Participants will be interviewed by the researcher using a validated, structured questionnaire. The questionnaire will be piloted on 10 patients who will be attending the endocrinology clinic. Patients usually attend the clinic once in two months after stabilization of their glycemic levels to normal value.

This structured data collection sheet (Annexure V) has three major parts. Demographic detail including age, gender, weight, height, etc., diabetes management including anti-diabetes drugs, investigation reports of FBS, HbA1c, total cholesterol, LDL, Triglyceride (TG) adverse effects and DM complications and diabetic knowledge assessment including attitude towards DM management and adherence to medication, knowledge about complications of DM, lifestyle habits, dietary control and physical exercise. Assessment of diabetes knowledge will be completed by the self-administered questionnaire and demographic and management of diabetes will be completed by extracting retrospective data from their clinical record books, investigation reports and drug files. Fasting blood sugar levels will be extracted at the time of diagnosis, 3/12 visit, 6/12 visit, 1 year and 2 years.

**Sample size**

The sample size (n) will be estimated in this cross-sectional study by using this formula,

n = (Z_(1-α)_ ) x ( p (1-p) )

D^2^

α = 0.05, Z_(1-α)_ = Z0.95 = 1.96, p = prevalence of DM in north central province in Sri Lanka is 9.6%, D = absolute precision and is taken as 0.04. Estimated total sample size according to the above equation is 208. Adding 5% of non-response rate, the sample size for each group (good glycemic control group and bad glycemic control group) will be 218 and the total sample size for this study will be 436 patients with type 2 diabetes.

For objective 2 and 3, the total sample will be divided in to two groups as good glycemic control (According to the guideline issued by SLCE Glycemic targets are, HbA1c<7.0% / FBS<130mg/dL / and PPBS<180mg/dL) and bad glycemic control (HbA1c>7.0% / FBS>130mg/dL / and PPBS >180mg/dL). For this, each group need 218 samples and the total sample size will be 436. To compare the efficacy of monotherapy vs early multidrug therapy to achieve glycemic targets in objective 1, the total sample will be divided into monotherapy and multidrug therapy. For objective 4, a total sample will be divided into groups according to the common combinations of oral hypoglycemic drugs used in the clinic (eg: Metformin and Gliclazide / Metformin and Tolbutamide, etc)

**Statistical analysis.**

Data will be analyzed by using SPSS V14 (SPSS Inc., Chicago, IL, USA) statistical software package. Data will be expressed as mean value +_SD for continuous variables. Frequency and proportions (%) will be reported for categorical variables. χ^2^ test will be used for comparing means and a student paired t-test will be used for comparing means between two groups. In all statistical analyses p<0.05 will be considered for significant differences.

For objective 1, paired t-test will be used and calculated mean values and P-values between monotherapy and multidrug therapy will be compared. A paired t-test will be applied for the initial blood sugar level and the blood sugar value in the last clinic visit, in the monotherapy group and multidrug therapy groups separately. And p-values will be compared. Also to understand the trend of blood sugar change during the clinic period in the monotherapy group and multidrug therapy group path analysis will be used.

For objective 4, the sample size will be divided into commonly use different drug combinations. A paired t-test will be applied for the initial blood sugar level and the blood sugar value in the last clinic visit. The p-values will be compared. Also, path analysis will be used to determine blood sugar levels during the clinic period. In addition to that, the effect of confounding factors will be matched in this statistical analysis.

Study plan

| Month | 2(Feb)  2018 | 3 | 4 | 5 | 6 | 7 | 8 | 9 | 10 | 11 | 12 | 1(Jan)  2019 | 2 | 3 | 4 | 5 | 6 | 7 | 8 |
| --- | --- | --- | --- | --- | --- | --- | --- | --- | --- | --- | --- | --- | --- | --- | --- | --- | --- | --- | --- |
| Submission of proposal | √ |  |  |  |  |  |  |  |  |  |  |  |  |  |  |  |  |  |  |
| PGIM approval |  | √ | √ | √ | √ |  |  |  |  |  |  |  |  |  |  |  |  |  |  |
| Ethics |  |  |  |  | √ | √ |  |  |  |  |  |  |  |  |  |  |  |  |  |
| Initial data collection |  |  |  |  |  | √ | √ | √ | √ | √ | √ | √ | √ |  |  |  |  |  |  |
| Writing dissertation |  |  |  |  |  |  |  |  |  |  |  |  | √ | √ | √ | √ | √ | √ |  |
| Submission |  |  |  |  |  |  |  |  |  |  |  |  |  |  |  |  |  |  | √ |

**Data collection sheet**

**Hospital Code:………….**

Started with (mono-therapy / Multi drug ) study ID …….

Date of data collection: …./…/2018 Age:………….Gender (Male/ Female)

Telephone No:…………………………………………………………

***Demographic***

Present Weight:……….kg. Height:…………cm BMI:……….kg/m^2^

Level of education: No formal education/ Primary education/ Secondary education/ Tertiary education

Occupation: ……………………………………………………………………………………………………..

Alcoholic ( ) Smoking ( )

Monthly income:……………………………………………………………………………………………….

Diagnosis: Type 2 DM/ Hypertension/Hyperlipidemia/other………………………………………………………

Date of diagnosis:…../…./201…

**Table 1: Glycemic control:**

|  | **At the time of diagnosis** | **3 months after diagnosis** | **6 months after diagnosis** | **1 year after diagnosis** | **2 years after diagnosis** |
| --- | --- | --- | --- | --- | --- |
| FBS |  |  |  |  |  |
| HbA1C |  |  |  |  |  |
| PPBS  ***Management of diabetes*** |  |  |  |  |  |
| OTT |  |  |  |  |  |
| Other investigations |  |  |  |  |  |
| Microalbuminuria |  |  |  |  |  |
| S. Creatinine |  |  |  |  |  |
| eGFR |  |  |  |  |  |
| **Drug treatment/dose** |  |  |  |  |  |
| Biguanide |  |  |  |  |  |
| Sulphonylureas |  |  |  |  |  |
| Acarbose |  |  |  |  |  |
| Thiazolidinediones |  |  |  |  |  |
| Other hypoglycemic drugs |  |  |  |  |  |
| Insulin |  |  |  |  |  |
| Weight |  |  |  |  |  |

**Table 2: Blood pressure control:**

|  | **At the time of diagnosis** | **3 months after diagnosis** | **6 months after diagnosis** | **1 year after diagnosis** | **2 years after diagnosis** |
| --- | --- | --- | --- | --- | --- |
| BP |  |  |  |  |  |
| Pulse |  |  |  |  |  |
| **Drug treatment** |  |  |  |  |  |
| Beta-blockers |  |  |  |  |  |
| ACEI |  |  |  |  |  |
| Ca channel blockers |  |  |  |  |  |
| Diuretics |  |  |  |  |  |
|  |  |  |  |  |  |
|  |  |  |  |  |  |

**Table 3: Lipid control:**

|  | **At the time of diagnosis** | **3 months after diagnosis** | **6 months after diagnosis** | **1 year after diagnosis** | **2 years after diagnosis** |
| --- | --- | --- | --- | --- | --- |
| Total cholesterol |  |  |  |  |  |
| LDL |  |  |  |  |  |
| HDL |  |  |  |  |  |
| TG |  |  |  |  |  |
| SGPT |  |  |  |  |  |
| SGOT |  |  |  |  |  |
| **Treatment** |  |  |  |  |  |
| Statin |  |  |  |  |  |
| Fibrates |  |  |  |  |  |
| Bile acid-binding resin |  |  |  |  |  |
| Other |  |  |  |  |  |

Other medications: Aspirin/……………………………/………………………./……………………/………………..

**Non-pharmacological management**

***Physical activities***

| **Physical activity** | **Frequency per week** |
| --- | --- |
| Walking |  |
| Cycling |  |
| Jogging |  |
| Manual labour |  |

***Dietary control***: (homemade/ outside)

| **Food** | **Frequency per week** |
| --- | --- |
| Vegetables in main meals |  |
| Fibres in the meal |  |
| Oily foods |  |
| Sweets |  |
| starchy foods |  |

| **Note:……………………………………………………………………………………………………………………………………………………………..……………………………………………………………………………………………………………………………………………………………………..……………………………………………………………………………………………………………………………………………………………………..………………………………………………………………………………** |
| --- |

**Table 4: Adverse drug reactions:**

| **Type of adverse effect** | **At the onset of treatment** | **Over the 2 years** | **At present** |
| --- | --- | --- | --- |
| ***For oral Hypoglycemic*** |  |  |  |
| **GI side effects**  **Nausea**  **Vomiting**  **Diarrhoea**  **Anorexia**  **Bloating sensation**  **Metallic taste** |  |  |  |
| **Weight gain (in kg)** |  |  |  |
| **Blood dyscrasias** |  |  |  |
| **Lactic acidosis** |  |  |  |
| **Allergy (At any time)** |  |  |  |
| **Hypoglycemia**  **Frequency**  **Severity:**  **Mild: below 70 mg/dL**   - Intense hunger - nervousness - shakiness - sweating   **Moderate: below 55 mg/dL**   - dizziness - sleepiness - confusion - difficulty in speaking   **Severe: below 35-40 mg/dL**   - seizure or convulsion - loss of consciousness - coma |  |  |  |
| **Other** |  |  |  |
| ***For Insulin*** |  |  |  |
| **Hypersensitivity reactions** |  |  |  |
| **lipohypertrophy**   - the elevated and thickened injection area - “rubbery feeling” - Painless   **Lipoatrophy** |  |  |  |
| **Other** |  |  |  |

**Complications at presentation.**

- **Microvascular complications:**
- Retinopathy (Yes/ No)
- Pathological changes in retina (including macula)

(Background retinopathy/ Pre-proliferative retinopathy/Proliferative retinopathy/Advanced retinopathy)

- Neuropathy (Yes/ No)

|  |
| --- |
|  |
|  |
|  |

- Peripheral neuropathy (Yes/No)
  - Numbness or loss of sensation (foot/hand)
  - Tingling or burning sensation(foot/hand)
  - Sharp pain or cramps(foot/hand)
  - Ulcers in foot or hands

|  |
| --- |
|  |
|  |

- Autonomic neuropathy
  - Impotence
  - gustatory sweating
  - postural hypotension
  - Other…………………………………………………………………………………………………..
- Diabetic amyotrophy
  - Severe pain in hip/thigh/ buttock
  - Difficulty in standing up from a sitting position
  - Other……………………………………………………………………………………………………..
- Mononeuropathy
- Nephropathy (Yes/ No)

|  |
| --- |
|  |
|  |

- - Microalbuminurea
  - eGFR <30ml/min/1.73m^2^
  - eGFR 30ml- 45ml/min/1.73m^2^
- **Macrovasculer complications:**
- Ischemic heart disease (Yes/ No)

(Acute MI-(ST elevation/non ST elevation)/unstable angina/stable angina)

- Peripheral vascular disease (Yes/ No/ don`t know)

(……………………………………………………………………………………………………)

- Cerebrovascular disease (Yes/ No)

(Stroke/TIA………………………………………………………………………………………………)

- **Metabolic complications**
- Hyperglycemia (Yes/ No)

|  |
| --- |
|  |
|  |

Severity

- - RBS>250mg/dL
  - Diabetes keto acidosis (Anion gap ≥12mEq/L + urine ketones)
  - Hyperglycemic hyperosmolar state (RBS≥600mg/dL)
- Infections (Skin/ GI tract/ Urinary tract/ Lungs)

**References**

1. Poretsky L, editor. Principles of Diabetes Mellitus [Internet]. Boston, MA: Springer US; 2004 [cited 2018 Feb 25]. Available from: http://link.springer.com/10.1007/978-1-4757-6260-0

2. WHO. WHO | Diabetes. WHO [Internet]. 2017 [cited 2018 Feb 17]; Available from: http://www.who.int/mediacentre/factsheets/fs312/en/

3. Wild S, Roglic G, Green A, Sicree R, King H. Global prevalence of diabetes: estimates for the year 2000 and projections for 2030. Diabetes Care [Internet]. 2004 May [cited 2018 Feb 17];27(5):1047–53. Available from: http://www.ncbi.nlm.nih.gov/pubmed/15111519

4. American Diabetes Association. Standards Of Medical Care In Diabetes 2015. J Clin Appl Res Educ. 2015;38(Supplement 1).

5. 8. Pharmacologic Approaches to Glycemic Treatment. Diabetes Care [Internet]. 2017 [cited 2018 Feb 25];40(40):64–74. Available from: https://pdfs.semanticscholar.org/39f0/318b8a446ff86e1e1968b392a6c872ec9aa6.pdf

6. Wong MCS, Kong APS, So W-Y, Jiang JY, Chan JCN, Griffiths SM. Adherence to oral hypoglycemic agents in 26 782 Chinese patients: A cohort study. J Clin Pharmacol. 2011;

7. Rwegerera GM. Adherence to anti-diabetic drugs among patients with Type 2 diabetes mellitus at Muhimbili National Hospital, Dar es Salaam, Tanzania-A cross-sectional study. 2014 [cited 2018 Feb 17]; Available from: https://www.ncbi.nlm.nih.gov/pmc/articles/PMC4189860/pdf/PAMJ-17-252.pdf

8. Stratton IM, Adler AI, Neil AW, Matthews DR, Manley SE, Cull CA, et al. Association of glycaemia with macrovascular and microvascular complications of type 2 diabetes (UKPDS 35): a prospective observational study. [cited 2018 Feb 17]; Available from: https://www.ncbi.nlm.nih.gov/pmc/articles/PMC27454/pdf/405.pdf

9. Jayawardena R, Ranasinghe P, Byrne NM, Soares MJ, Katulanda P, Hills AP. Prevalence and trends of the diabetes epidemic in South Asia: a systematic review and meta-analysis. [cited 2018 Feb 17]; Available from: https://bmcpublichealth.biomedcentral.com/track/pdf/10.1186/1471-2458-12-380?site=bmcpublichealth.biomedcentral.com

10. Katulanda P, Rathnapala DA V, Sheriff R, Matthews DR. Province and ethnic-specific prevalence of diabetes among Sri Lankan adults. Sri Lanka J Diabetes Endocrinol Metab [Internet]. 2011 [cited 2018 Feb 17];1:2–7. Available from: file:///C:/Users/admin/Downloads/4180-14954-1-PB.pdf

11. Amarasekara AA, Fongkaew W, Wimalasekera SW, Turale S, Chanprasit C. Cross-sectional study of glycemic control among adults with type 2 diabetes. Nurs Heal Sci. 2015;

12. Finnish Diabetes Prevention Study G. Prevention in Type 2 Diabetes mellitus by changes in lifestyle among subjects with impaired glucose tolerance. N Engl J Med. 2001;

13. Abhayaratna SA, Somaundaram NP, Rajapakse H. Prevalence of the metabolic syndrome among patients with type 2 diabetes. Sri Lanka J Diabetes Endocrinol Metab [Internet]. 2015 [cited 2018 Feb 17];795(5):79–84. Available from: file:///C:/Users/admin/Downloads/7286-25744-1-SM.pdf

14. Weerasuriya N, Siribaddana2 S, Dissanayake A, Subasinghe3 Z, Wariyapola3 D, Fernando DJS. Long-term complications in newly diagnosed Sri Lankan patients with type 2 diabetes mellitus. Q J Med [Internet]. 1998 [cited 2018 Feb 17];91:439–43. Available from: http://citeseerx.ist.psu.edu/viewdoc/download?doi=10.1.1.521.9420&rep=rep1&type=pdf

15. Somasundaram NP, Wijeyaratne CN, De Silva S, Siribaddana S, Illangasekera U, Abhayaratna S, et al. Diabetes Mellitus: Glucose Control Clinical guidelines: The Endocrine Society of Sri Lanka. Sri Lanka J Diabetes Endocrinol Metab. 2013;
